# Characteristics of Patients with Oseltamivir-Resistant Pandemic (H1N1) 2009, United States

**DOI:** 10.3201/eid1702.101724

**Published:** 2011-02

**Authors:** Samuel B. Graitcer, Larisa Gubareva, Laurie Kamimoto, Saumil Doshi, Meredith Vandermeer, Janice Louie, Christine Waters, Zack Moore, Katrina Sleeman, Margaret Okomo-Adhiambo, Steven A. Marshall, Kirsten St. George, Chao-Yang Pan, Jennifer M. LaPlante, Alexander Klimov, Alicia M. Fry

**Affiliations:** Author affiliations: Centers for Disease Control and Prevention, Atlanta, Georgia, USA (S.B. Gratcer, L. Gubareva, L. Kamimoto, S. Doshi, K. Sleeman, M. Okomo-Adhiambo, A. Klimov, A.M. Fry);; Oregon Department of Human Services, Portland, Oregon, USA (M. Vandermeer);; California Department of Health, Sacramento, California, USA (J. Louie, C.-Y. Pan);; New York State Department of Health, Albany, New York, USA (C. Waters, K. St. George, J.M. LaPlante);; North Carolina Department of Health and Human Services, Raleigh, North Carolina, USA (Z. Moore);; Wisconsin Department of Health, Madison, Wisconsin, USA (S.A. Marshall)

**Keywords:** Oseltamivir, influenza, antimicrobial resistance, influenza A, H1N1 subtype, viruses, pandemic (H1N1) 2009, United States, expedited, dispatch

## Abstract

During April 2009–June 2010, thirty-seven (0.5%) of 6,740 pandemic (H1N1) 2009 viruses submitted to a US surveillance system were oseltamivir resistant. Most patients with oseltamivir-resistant infections were severely immunocompromised (76%) and had received oseltamivir before specimen collection (89%). No evidence was found for community circulation of resistant viruses; only 4 (unlinked) patients had no oseltamivir exposure.

During April, 2009**–**June, 2010 the United States had enhanced surveillance for oseltamivir resistance among pandemic influenza A (H1N1) 2009 viruses. We describe characteristics of patients infected with oseltamivir-resistant and oseltamivir-susceptible pandemic (H1N1) 2009 virus.

## The Study

During April 2009**–**June 2010, the Centers for Disease Control and Prevention (CDC) requested state public health laboratories to submit specimens for antiviral susceptibility testing by 2 routes. In the first route, the first 5 influenza specimens of any type or subtype collected every 2 weeks from each laboratory underwent virus isolation for comprehensive antiviral testing, including testing by neuraminidase inhibition (NI) assay, sequencing viruses with elevated 50% inhibitory concentration (IC_50_) values, and pyrosequencing for adamantine resistance–conferring M2 mutations. In the second route, the first 5 additional clinical specimens from pandemic (H1N1) 2009 virus–infected patients that were collected each week by these laboratories were submitted and screened for the oseltamivir-resistant conferring neuraminidase H275Y mutation by using pyrosequencing. Patients with oseltamivir-resistant pandemic (H1N1) 2009 infection had demographic and clinical information collected by using a standard form.

Oseltamivir resistance was determined by either NI or pyrosequencing for the H275Y mutation. NI was performed on virus isolates with a chemiluminescent substrate; viruses with elevated IC_50_ values for oseltamivir were identified as resistant, based on previously set criteria (*1**,**2*). All oseltamivir-resistant viruses had H275Yconfirmed by pyrosequencing (*1*). Original clinical specimens collected from surveillance were screened by pyrosequencing for H275Y, without NI. NI testing was performed at CDC, and pyrosequencing for H275Y was performed at CDC and state laboratories in Wisconsin, New York, and California. All oseltamivir-resistant viruses referenced here were reported on FluView (*3*). Four patients, identified in June and August 2009, were reported previously (*4**,**5*).

A comparison group of hospitalized patients infected with oseltamivir-susceptible pandemic (H1N1) 2009 was identified from the Influenza Hospitalization Network (FluSurv-NET). FluSurv-NET includes 10 states that participate in the Emerging Infections Program, a population-based surveillance for hospitalized patients with influenza infection (California, Colorado, Connecticut, Georgia, Maryland, Minnesota, New Mexico, New York, Oregon, Tennessee), plus 6 states (Iowa, Idaho, Michigan, North Dakota, Oklahoma, South Dakota) added in response to the 2009 pandemic, as previously described (*3**,**6*). The counties within FluSurv-NET represent 26 million persons (8.5% of the US population) (*6*). The 16 states participating in FluSurv-NET collected demographic and clinical information for all hospitalized patients with laboratory-confirmed influenza infection within their catchment counties (*6*). We identified patients hospitalized in FluSurv-NET who had specimens submitted to national antiviral resistance surveillance by using Link Plus software to link antiviral resistance surveillance and FluSurv-NET data by patient county of residence, age, and sex and specimen collection date.

We considered identical matches on all 4 variables as a high probability match, e.g., a patient from FluSurv-NET who had a pandemic (H1N1) 2009 virus specimen submitted to national antiviral resistance surveillance who had an oseltamivir-susceptible pandemic (H1N1) 2009 virus infection. We validated our linking methods with Oregon data (n = 41); all 4 patients identified as high probability matches were true matches. For validation purposes, we identified 4 specimens that were matched on county, age, and sex but not on specimen collection date up to 7 days, e.g., moderate probability matches; 1 patient was hospitalized, 2 were outpatients, and 1 specimen was from a medical examiner (patient not hospitalized). The Oregon surveillance specimens that were neither high nor moderate probability matches were surveillance specimens from outpatients and cluster investigations (M. Vandermeer, pers. comm.).

Overall, 6,740 virus isolates and specimens were submitted to surveillance systems; 37 (0.5%) viruses were oseltamivir resistant (*3*); 18 were identified by NI, contained the H275Y mutation, and were susceptible to zanamivir and resistant to adamantanes; the 19 remaining viruses were detected by pyrosequencing for H275Y. Oseltamivir-susceptible viruses exhibited IC_50_ values ranging from 0.05 to 1.44 nmol/L. Oseltamivir-resistant viruses exhibited a median IC_50_ value of 80.08 nmol/L (range 6.24–116.48 nmol/L).

Most patients infected with oseltamivir-resistant pandemic (H1N1) 2009 viruses were hospitalized (81%), had a severe immunocompromising condition (76%), and had been exposed to oseltamivir before collection of the specimen tested for antiviral resistance (89%) (Table); 9 (30%) had received oseltamivir as chemoprophylaxis, and 21 (70%) had received oseltamivir as treatment. Four patients with oseltamivir-resistant pandemic (H1N1) 2009 virus infection had no documented exposure to oseltamivir before collection of the specimen for testing, including exposure to family members receiving oseltamivir. No epidemiologic links were found between the 4 patients.

**Table:** Characteristics of patients infected with oseltamivir-resistant and -susceptible pandemic (H1N1) 2009 viruses from national influenza antiviral resistance surveillance and enhanced hospital influenza surveillance, April 2009–June 2010*†

Characteristic	Oseltamivir-resistant infections			Oseltamivir-susceptible infections	
	Total from national surveillance, n = 37	Total from FluSurv-NET states,‡ n = 17		National surveillance cases from FluSurv-NET counties, n = 401	National surveillance cases matched in FluSurv-NET, n = 65
Median age, y (range)	18 (1–74)	21 (5–74)		22 (0–89)	31 (0–82)
Female sex	18 (49)	6 (35)		177 (50)	38 (58)
Hospitalized	30 (81)	16 (100)		65 (16)	65 (100)
ICU admission	13/30 (43)	7/12 (58)		–	14 (23)
Deaths, all cause§	7/31 (23)	2/12 (17)		–	3 (5)
Oseltamivir exposure¶	31 (89)	16 (94)		–	6 (14)
Underlying medical condition	33 (89)	17 (100)		–	49 (75)
Severe/Immunosuppression#	28 (76)	17 (100)		–	7 (11)**
Pregnancy	1 (2.7)	0		–	5 (7.7)
Asthma and CLD	10 (27)	2 (18)		–	21 (34)
CVD	8 (22)	4 (24)		–	5 (8)
Diabetes mellitus	5 (14)	2 (13)		–	12 (18)
Chronic kidney disease	4 (11)	3 (19)		–	5 (8)
No underlying medical conditions	4 (11)	0		–	13 (21)
Median time from oseltamivir initiation to specimen collection, d (range)	11 (2–37)	14 (3–37)		–	1.5 (1–4)††

*Values are no. (%) except as indicated. FluSurv-NET, Influenza Hospitalization Network; ICU, intensive care unit; CLD, chronic lung disease; CVD, cardiovascular disease, excluding hypertension; –, not applicable. †Missing data were excluded from analysis; denominators are included where they varied from cohort size. ‡Of the 16 oseltamivir-resistant cases from states participating in FluSurv-NET, 5 had county information and were from FluSurv-NET counties. None were high probability matches to a FluSurv-NET hospitalized patient and none were from Oregon. FluSurv-NET captured only 1 hospitalization for each patient infected with pandemic (H1N1) 2009 virus during the year. Repeat hospitalizations were not recorded, although many sites noted repeat hospitalizations for patients with immunosuppressive conditions (L. Kamimoto, pers. comm.). §Among oseltamivir-resistant cases, 4 deaths were reported to national surveillance as directly caused by influenza. Cause of death was not recorded for patients infected with oseltamivir-susceptible pandemic (H1N1) 2009 virus. ¶Patients with oseltamivir exposure received oseltamivir, either as chemoprophylaxis or treatment, before the collection date of the pandemic (H1N1) 2009 virus specimen tested for antiviral resistance. #For patients with oseltamivir-resistant pandemic (H1N1) 2009, severe immunosuppression was defined as any of the following: receiving treatment for any cancer within 6 months before onset of influenza illness, currently receiving immunosuppressive medication, including systemic corticosteroids, as part of prevention strategies for transplant (bone marrow or solid organ) rejection, or for management of pulmonary or autoimmune conditions, or having a diagnosis of AIDS, not just HIV infection. For patients within FluSurv-NET, we included any patient with a medical record of the syndromes above or if immunosuppressed or immunosuppression was recorded in the medical chart. **Among the 7 hospitalized patients from FluSurv-NET with oseltamivir-susceptible pandemic (H1N1) 2009 and an immunosuppressive condition, 3 (43%) were receiving chronic systemic corticosteroids; 1 for systemic lupus erythematosus and the other 2 for unknown reasons. The immunosuppressive condition was not known for 4 patients, but immunosuppression was recorded from the medical record. ††n = 6.

Among the 28 patients infected with oseltamivir-resistant pandemic (H1N1) 2009 virus, with a severe immunocompromising condition and a complete case form, 24 (86%) had a malignancy reported, 23 had a hematologic malignancy and were receiving chemo- or immunosuppressive therapy at the time of their infection, and 10 (38%) were recipients of a hematopoietic stem cell transplant (SCT). One patient had AIDS and a lymphoma of the central nervous system. Among the 3 immunosuppressed patients without a malignancy, 2 were recipients of solid organ (renal) transplants, and another had received SCT <6 months before influenza illness (reason for SCT could not be confirmed).

Among 1,982 national surveillance oseltamivir-susceptible specimens from the 16 FluSurv-NET states, 1,607 (81%) had county information; among these, 401 (25%) specimens were from FluSurv-NET counties, and 65 patients from FluSurv-NET were high probability matches to patients identified in antiviral resistance surveillance data (Table). Compared with patients with oseltamivir-resistant pandemic (H1N1) 2009 infections identified in national surveillance, few (11%) FluSurv-NET patients with an oseltamivir-susceptible pandemic (H1N1) 2009 virus infection had severely immunosuppressive conditions, and few (14%) had oseltamivir exposure before collection of the specimen for testing, none were reported to have received oseltamivir as chemoprophylaxis. Among all 8,740 FluSurv-NET hospitalized patients with pandemic (H1N1) 2009 during this period, 10% had an immunosuppressive condition. Patients with oseltamivir-resistant infections had specimens for testing collected a median of 11 (range 2–37) days after oseltamivir initiation, and results may reflect testing due to clinical suspicion of resistance. Among the 6 FluSurv-NET patients with specimens collected after oseltamivir was begun, the median time between oseltamivir initiation and specimen collection was shorter.

## Conclusions

Infections with oseltamivir-resistant pandemic (H1N1) 2009 viruses were rare in the United States during April 2009**–**June 2010. Few patients had no oseltamivir exposure before resistant virus was detected, and none had epidemiologic links to another patient. Thus, evidence for community transmission of oseltamivir-resistant pandemic (H1N1) 2009 viruses was rare (*7*). Patients with severe immunocompromising conditions with prior exposure to oseltamivir were most likely to have an oseltamivir-resistant infection. Infections were most frequently reported in patients with hematologic cancers who were undergoing immunosuppressive treatment, chemotherapy, or SCT. Other studies have also reported a high frequency of patients with hematologic malignancies or SCT and oseltamivir exposure among patients with oseltamivir-resistant pandemic (H1N1) 2009 virus infections (*8**,**9*). Oseltamivir resistance should be considered among patients with severe immunocompromising conditions and pandemic (H1N1) 2009 in the setting of oseltamivir treatment or chemoprophylaxis failure.

Although the number of patients with oseltamivir-resistant pandemic (H1N1) 2009 virus infections was small in the United States during this period, this is the largest case series published and confirms findings from reports with smaller samples (*8**–**10*). Although all patients in our comparison group of patients with oseltamivir-susceptible pandemic (H1N1) 2009 were hospitalized, most patients in the oseltamivir-resistant group were also hospitalized. Finally, we do not have a comparison group of patients with immunocompromising conditions and oseltamivir-susceptible pandemic (H1N1) 2009 virus infections; thus, risk factors for infection with oseltamivir-resistant infection among patients with immunocompromising conditions cannot be determined. The finding of oseltamivir-resistant pandemic (H1N1) 2009 viruses associated with oseltamivir treatment highlights the need for new antiviral agents and new treatment strategies.
